# A Rare Triad of Type 1 Distal Renal Tubular Acidosis, Cryptogenic Multifocal Ulcerous Stenosing Enteritis, and Superior Mesenteric Artery Syndrome in a Young Adult

**DOI:** 10.7759/cureus.97993

**Published:** 2025-11-28

**Authors:** Neha Shehzad, Viswesh Anand, Karan Aggarwal, Salah-ud-din Taj

**Affiliations:** 1 Acute Medicine, United Lincolnshire Teaching Hospitals NHS Trust, Lincoln, GBR; 2 Internal Medicine, University Hospitals of Derby and Burton, Derby, GBR; 3 Internal Medicine, United Lincolnshire Teaching Hospitals NHS Trust, Lincoln, GBR

**Keywords:** cryptogenic multifocal ulcerous stenosing enteritis, hypokalaemia, renal tubular acidosis, superior mesenteric artery syndrome, weight loss and malnutrition

## Abstract

Recurrent hypokalaemia and iron-deficiency anaemia in a young adult should prompt evaluation for uncommon renal and gastrointestinal disorders. The coexistence of distal renal tubular acidosis (dRTA), cryptogenic multifocal ulcerous stenosing enteritis (CMUSE), and superior mesenteric artery (SMA) syndrome is exceptionally rare. A woman in her 30s presented in 2019 with profound hypokalaemia and cardiac arrest secondary to possible dRTA. Biochemistry confirmed non-anion gap metabolic acidosis with inappropriate renal potassium loss. Despite supplementation, she later developed severe iron-deficiency anaemia and underwent small-bowel resection for capsule retention; histology confirmed CMUSE. Subsequent weight loss and malnutrition led to radiological evidence of SMA syndrome. She experienced recurrent admissions for electrolyte crises, requiring long-term intravenous replacement via a peripherally inserted central catheter and multidisciplinary input from nephrology, gastroenterology, and endocrinology. This case illustrates the cumulative burden and interdependence of multiple rare disorders within one patient, emphasising the need for persistent diagnostic review, multidisciplinary care, and awareness of atypical disease combinations in young adults with refractory electrolyte and nutritional abnormalities.

## Introduction

Hypokalaemia and iron-deficiency anaemia are frequently encountered in clinical practice; their repeated occurrence in a young adult should raise suspicion for less common renal or gastrointestinal disorders. Distal renal tubular acidosis (dRTA) is rare, with population-based studies estimating a prevalence of 0.46-1.6 per 10,000 individuals [1]. Cryptogenic multifocal ulcerous stenosing enteritis (CMUSE) is also extremely uncommon, with approximately 200 cases reported worldwide [2]. Superior mesenteric artery (SMA) syndrome, although more recognised, remains rare, with an estimated incidence of 0.1%-0.3% in the general population [3]. Both dRTA and CMUSE are uncommon in isolation [4-9], and the additional development of SMA syndrome in the same patient represents an exceptional constellation of pathologies [10-12].

## Case presentation

A woman in her 30s was first admitted in 2019 with fatigue, dyspnoea, and altered consciousness. She was found to have profound hypokalaemia and metabolic acidosis, and shortly after admission, sustained a pulseless electrical activity (PEA) cardiac arrest. She was resuscitated and admitted to the ICU, receiving IV electrolyte correction. Biochemistry demonstrated hypokalaemia, low bicarbonate, non-anion gap metabolic acidosis, preserved renal function, and elevated urinary potassium, features suggestive of distal RTA. Initial workup (autoimmune, paraproteinemia, heavy metals) was negative. She stabilised on potassium citrate and sodium bicarbonate. Serial biochemical parameters during follow-up are summarised in Table 1.

**Table 1:** Serial laboratory values, interpretation: persistent hypokalaemia with inappropriate renal potassium loss, normal renal function, and low bicarbonate → consistent with possible distal RTA. All diagnostic investigations and biochemical parameters reported were standard clinical tests freely used in routine care; no licensed tools were required. RTA: renal tubular acidosis, IDA: iron-deficiency anaemia.

Date	Serum K⁺ (mmol/L), Reference Range: 3.5-5.0 mmol/L	Serum Na⁺ (mmol/L), Reference Range: 135-145 mmol/L	Serum Mg²⁺ (mmol/L), Reference Range: 0.7-1.0 mmol/L	Random Urine K⁺ (mmol/L), Reference Range: < 20 mmol/L	Hb (g/L), Reference Range: 120-160 g/L	Notes
Jul 2022	3.2	139	–	–	–	Low-normal K⁺
Sep 2022	3.4	141	0.78	–	–	
Sep 2023	2.4	149	1.41	96	66-95	Severe hypokalaemia, IDA
Aug 2024	3.4	140	0.84	25	–	
Jul 2025	2.7	144	0.69	38	–	Frequent IV replacement
Aug 2025	3.8	135	0.73	40	–	Post-IV correction

In 2021, she developed severe iron-deficiency anaemia (Hb, 53 g/L). Oesophago-gastro-duodenoscopy (OGD) showed hiatus hernia/mild gastritis; colonoscopy was normal. Capsule endoscopy was retained, leading to small bowel obstruction. She underwent laparoscopic resection of 30 cm ileum. Histology post-surgery confirmed CMUSE with multiple diaphragm-like strictures and fibrosis. A summary of histopathological findings and additional investigations is provided in Table 2.

**Table 2:** Summary of histopathology and other investigations. All diagnostic investigations and biochemical parameters reported were standard clinical tests freely used in routine care; no licensed tools were required.

Category	Findings
Histopathology (Ileum resection, 2021)	Histopathological examination of the resected ileal specimen revealed a 320 mm segment of small intestine containing four fibrotic, diaphragm-like strictures. Microscopy demonstrated shallow mucosal ulcerations, submucosal fibrosis, mixed inflammatory cell infiltration, and vascular congestion. There was no evidence of granulomas, Crohn’s-like transmural inflammation, vasculitis, cytomegalovirus (CMV) infection, dysplasia, or malignancy. The mesenteric lymph nodes were reactive (0/5). Overall, these findings were strongly supportive of a diagnosis of cryptogenic multifocal ulcerous stenosing enteritis (CMUSE).
Urine studies	Elevated urinary potassium and potassium:creatinine ratio > 4.
Imaging	CT abdomen: capsule retention and later superior mesenteric artery (SMA) syndrome [7-9].
Histology correlation	Presence of diaphragm-like strictures consistent with CMUSE [4-6].
Negative investigations	Coeliac serology, autoimmune screen, myeloma workup, and heavy-metal testing all negative.
Genetic testing	Panel for inherited tubulopathies negative [2].

**Figure 1:**
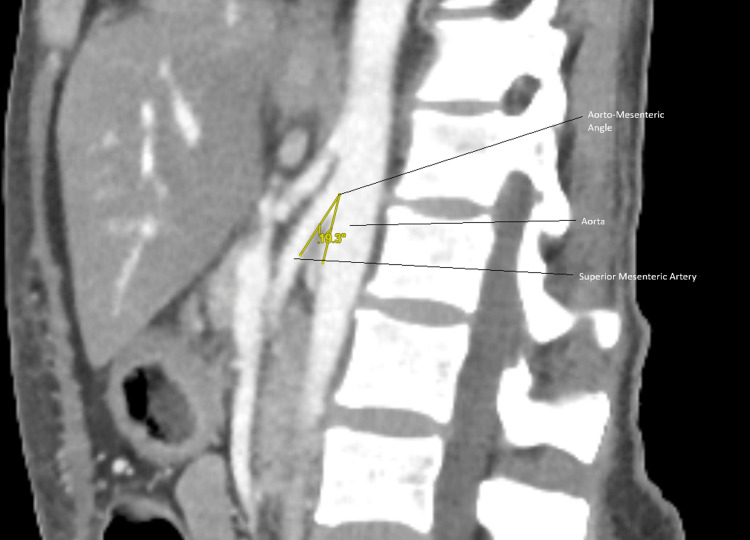
Sagittal contrast-enhanced CT showing a reduced aortomesenteric angle of 19.3°, below the diagnostic threshold of <22°, resulting in compression of the third part of the duodenum and confirming superior mesenteric artery syndrome.

**Figure 2:**
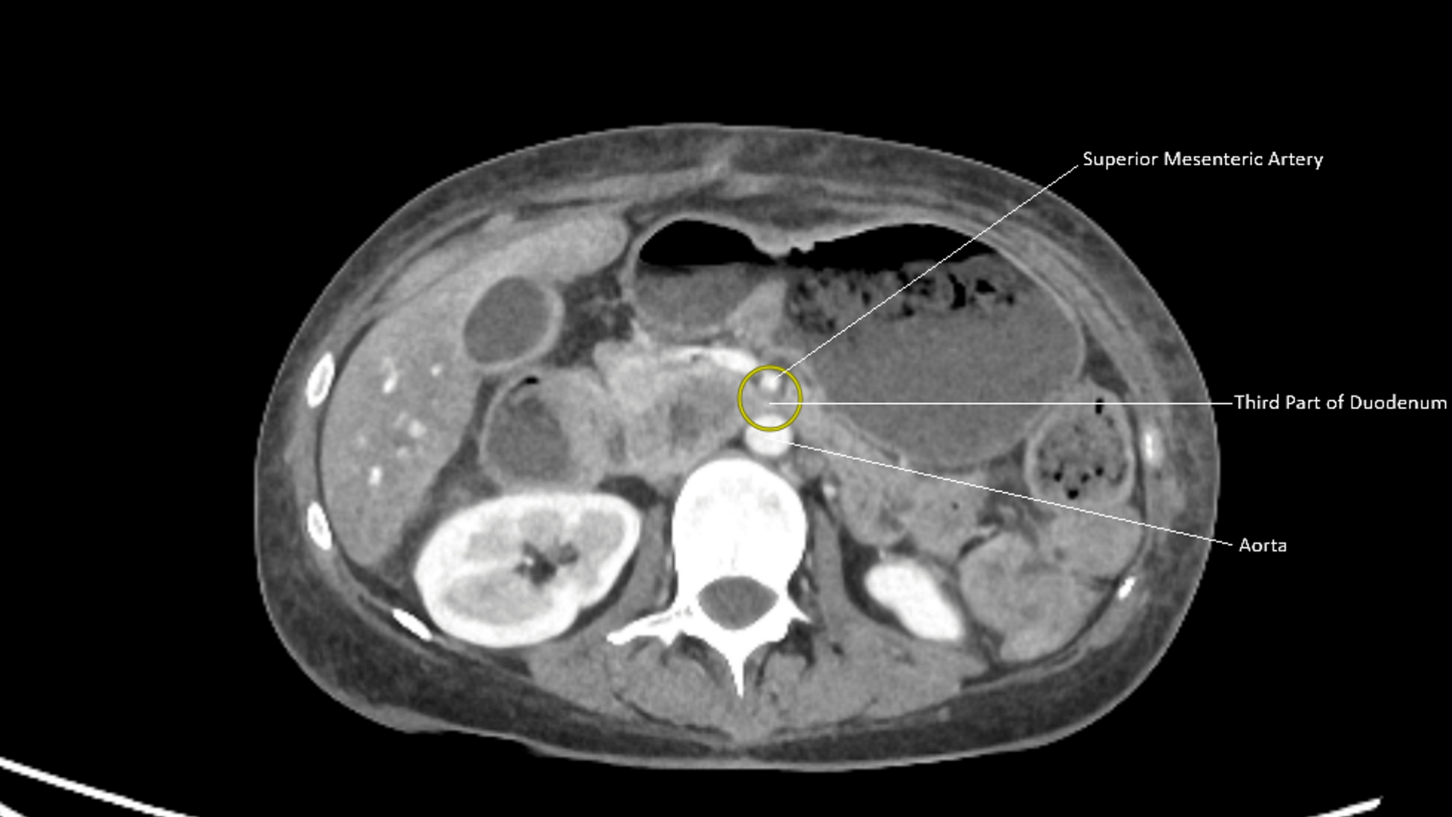
Axial CT showing compression of the third part of the duodenum between the superior mesenteric artery (SMA) and aorta, consistent with SMA syndrome.

In 2023, she developed septic shock from a dental abscess requiring extraction of upper right first molar (UR6)/upper right second molar (UR7) and washout. This was complicated by an oro-antral fistula, candidiasis, and worsening nutritional status. In 2024, CT abdomen demonstrated duodenal compression between the aorta and SMA, consistent with SMA syndrome, see Figures 1, 2. The sequence of radiological investigations and their findings is detailed in Table 3.

**Table 3:** Imaging timeline. All diagnostic investigations and biochemical parameters reported were standard clinical tests freely used in routine care; no licensed tools were required. SMA: superior mesenteric artery.

Date	Findings	Impression
Feb 2025	CT abdomen: free gas/fluid, bowel perforation; patchy lung consolidation.	Suspicious for bowel perforation.
May 2025	CT: Resolution of lung changes; duodenal compression between SMA and aorta.	SMA syndrome.
May 28, 2025	CT: Extensive inflammatory lung changes, pleural effusion, mediastinal nodes, anasarca.	Inflammatory/infective episode.
Jul 29, 2025	CT: Small bowel obstruction at prior anastomosis, stable liver cysts.	Small bowel obstruction, no perforation.

From 2024 to 2025, she experienced recurrent episodes of severe hypokalaemia (<2.5 mmol/L) requiring frequent IV correction in same-day emergency care (SDEC). Oral replacement was poorly tolerated. A peripherally inserted central catheter (PICC) line was inserted for regular infusions.

Differential diagnosis

The main differential diagnoses considered included hypokalaemia secondary to possible distal renal tubular acidosis versus gastrointestinal potassium loss, anaemia due to cryptogenic multifocal ulcerous stenosing enteritis (CMUSE) versus anaemia of chronic disease, and superior mesenteric artery (SMA) syndrome occurring either idiopathically or as a postoperative complication. These differentials were assessed through the integration of biochemical profiles, imaging findings, and the patient’s clinical course.

Treatment

Management comprised ongoing electrolyte replacement, surgical intervention, infection control, and nutritional optimisation. Electrolyte disturbances were addressed with oral and intravenous potassium, sodium bicarbonate, and magnesium supplementation, administered via a peripherally inserted central catheter (PICC) when required. Surgical procedures included ileal resection in 2021, dental extraction and washout in 2023, and an emergency laparotomy for bowel perforation in 2025. Infective complications such as streptococcal bacteraemia were treated with appropriate antimicrobial therapy. Nutritional management required continuous input from dietitians due to poor oral tolerance from SMA syndrome, with multidisciplinary follow-up involving nephrology, gastroenterology, and endocrine teams.

Outcome and Follow-Up

She continues to be under close nephrology, gastroenterology, and endocrine review. Potassium requires frequent monitoring and IV supplementation. Nutritional status remains fragile, with consideration for further intervention for SMA syndrome.

## Discussion

This case highlights the unusual coexistence of three rare disorders, possible distal renal tubular acidosis (dRTA), cryptogenic multifocal ulcerous stenosing enteritis (CMUSE), and superior mesenteric artery (SMA) syndrome, in the same patient, resulting in repeated hospitalisations, nutritional deterioration, and recurrent electrolyte crises.

Distal renal tubular acidosis and hypokalaemia

dRTA arises when the distal nephron is unable to adequately excrete hydrogen ions, producing a chronic metabolic acidosis with a normal anion gap. This defect drives urinary potassium wasting and persistent hypokalaemia, even when glomerular function is preserved [4]. Clinical consequences can range from tiredness and muscle weakness to paralysis or arrhythmias, and cardiac arrest has been described in severe cases [4,6].

In our patient, profound hypokalaemia and metabolic acidosis (pH, 7.21; reference range, 7.35-7.45) culminating in a pulseless electrical activity (PEA) arrest, together with supportive biochemical findings, pointed towards dRTA. Despite an extensive workup, including autoimmune, paraprotein, and toxicology screening as well as a negative genetic panel, no secondary aetiology was identified. The working diagnosis therefore remains “possible dRTA,” made on the basis of physiology and exclusion of other causes. The patient’s continued potassium wasting, coupled with intolerance of oral supplementation, necessitated ongoing IV therapy, demonstrating both the severity of her electrolyte disorder and the practical challenges of its management in the context of gastrointestinal comorbidity.

Cryptogenic multifocal ulcerous stenosing enteritis (CMUSE)

CMUSE is a very uncommon idiopathic disease of the small intestine, first reported in the 1960s. It is characterised by multiple diaphragm-like strictures and superficial mucosal ulcerations but differs from Crohn’s disease in that it lacks transmural inflammation or granulomas [7,8]. Patients typically present with recurrent obstruction, abdominal discomfort, and iron-deficiency anaemia.

In this case, CMUSE was diagnosed after capsule endoscopy retention led to surgical resection. Histology demonstrated classic features, including fibrotic strictures, superficial ulceration, and vascular congestion, while differential diagnoses such as Crohn’s disease, vasculitis, and non-steroidal anti-inflammatory drug (NSAID)-related enteropathy were excluded. The disease course is often progressive, with a tendency to recur despite surgical intervention. Although corticosteroid therapy has been attempted, responses are inconsistent and long-term outcomes remain unsatisfactory [9].

Superior mesenteric artery syndrome

SMA syndrome is a rare mechanical cause of duodenal obstruction, resulting from compression of the third part of the duodenum between the SMA and the aorta. It is most often seen following significant weight loss, spinal surgery, or severe catabolic states [10,11]. Patients usually report postprandial pain, nausea, and vomiting, with subsequent nutritional decline.

In our patient, SMA syndrome was identified on CT imaging after ileal resection and associated weight loss. The condition created a vicious cycle: worsening malnutrition reduced tolerance of oral supplements, which in turn perpetuated her electrolyte and nutritional instability. This highlights the complex interdependence between structural and functional gastrointestinal disease.

Interplay of pathologies

The concurrence of these three rare conditions in a single patient is remarkable, as each pathology exacerbated the others. Persistent hypokalaemia from suspected distal renal tubular acidosis was compounded by nutritional deficits and reduced oral intake. CMUSE contributed to chronic anaemia, malabsorption, and surgical morbidity, thereby setting the stage for the later development of superior mesenteric artery (SMA) syndrome. In turn, SMA syndrome further impaired tolerance to oral potassium and bicarbonate supplementation, resulting in a dependence on intravenous replacement. This interrelationship explains the recurrent hospitalisations and the difficulty in achieving long-term stability despite coordinated multidisciplinary care.

Comparison with published literature

Severe hypokalaemia leading to cardiac arrest in the context of distal renal tubular acidosis (dRTA) has been described previously [4,6], and Bhandarkar et al. [5] highlighted the broad clinical spectrum of renal tubular acidosis and the diagnostic challenges encountered when no secondary trigger is identified. Capsule retention due to small bowel strictures, as seen in our patient, has similarly been reported in cases of cryptogenic multifocal ulcerous stenosing enteritis (CMUSE) by Chen et al. [7] and Tao et al. [8], while Samanta et al. [9] emphasised the histopathological distinction of CMUSE from Crohn’s disease and gastrointestinal tuberculosis. Comparable postoperative and malnutrition-related presentations of superior mesenteric artery (SMA) syndrome have been documented by Agrawal et al. [10], White et al. [11], and Berken et al. [12]. To our knowledge, this is the first reported case combining possible dRTA, CMUSE, and SMA syndrome, illustrating the cumulative burden of multiple rare pathologies in a single patient.

Learning points

This case illustrates several important learning points. Persistent hypokalaemia with preserved renal function should prompt consideration of distal renal tubular acidosis. CMUSE should remain an important differential diagnosis in patients presenting with unexplained small bowel strictures and recurrent iron-deficiency anaemia. Postoperative SMA syndrome can perpetuate malnutrition and complicate management. Multidisciplinary collaboration is essential for optimal outcomes in complex multisystem cases, and a pragmatic working diagnosis may be necessary when exhaustive investigations yield no definitive cause.

## Conclusions

This report describes an exceptionally rare concurrence of distal renal tubular acidosis, cryptogenic multifocal ulcerous stenosing enteritis, and superior mesenteric artery syndrome within a single patient. The interaction between these disorders produced a complex clinical picture marked by recurrent electrolyte imbalance, malnutrition, and repeated hospital admissions. The case highlights the importance of sustained clinical vigilance and the value of revisiting differential diagnoses when standard investigations fail to explain persistent abnormalities. Coordinated multidisciplinary follow-up is crucial in managing such multifactorial conditions and ensuring long-term stability.
